# Association between intracranial and extracranial atherosclerosis and white matter hyperintensities: a systematic review and meta-analysis

**DOI:** 10.3389/fnagi.2023.1240509

**Published:** 2024-01-08

**Authors:** Wenyuan Zhang, Fangwang Fu, Zhenxiang Zhan

**Affiliations:** ^1^Department of Neurology, Affiliated Yueqing Hospital, Wenzhou Medical University, Yueqing, China; ^2^Department of Neurology, The Second Affiliated Hospital and Yuying Children’s Hospital of Wenzhou Medical University, Wenzhou, China; ^3^Department of Neurology, Affiliated Jinhua Hospital, Zhejiang University School of Medicine, Jinhua, China

**Keywords:** cerebral small vessel disease, extracranial atherosclerosis, intracranial atherosclerosis, meta-analysis, white matter hyperintensities

## Abstract

**Background:**

White matter hyperintensities (WMHs) are key neuroimaging markers of cerebral small vessel diseases. This study aimed to investigate whether intracranial and extracranial atherosclerotic stenosis is associated with WMHs.

**Methods:**

Following a previously registered protocol (PROSPERO protocol: CRD42023407465), PubMed, Web of Science, and Embase were systematically searched for relevant literature published until March 2023. Cross-sectional studies examining the association between intracranial and extracranial atherosclerotic stenosis and WMHs were included. Random effects models were used to calculate the pooled estimates.

**Results:**

Twenty-one eligible studies, including 10,841 participants, were identified. Intracranial and extracranial atherosclerotic stenosis was associated with an increased risk of WMHs (OR 1.80, 95% CI 1.25–2.57, *I*^2^ = 75%) and increased WMH volumes (SMD 0.40, 95% CI 0.18–0.63, *I*^2^ = 63%). Heterogeneity resulted from the WMHs rating method and the location. Extracranial atherosclerotic stenosis (ECAS) was significantly associated with WMHs (OR 2.10, 95% CI 1.22–3.62, *I*^2^ = 71%), but intracranial atherosclerotic stenosis (ICAS) was insignificantly associated with WMHs (OR 1.75, 95% CI 0.97–3.15, *I*^2^ = 84%). The association was stable in the subgroup analysis based on WMHs location, which included deep WMHs and periventricular WMHs.

**Conclusion:**

Intracranial and extracranial atherosclerotic stenosis is associated with WMHs. This association is significant in ECAS, but attenuated in ICAS.

## Introduction

White matter hyperintensities (WMHs) of presumed vascular origin are ischemic manifestations of cerebral small vessel disease that can be detected through neuroimaging (Wardlaw et al., 2013). The prevalence of WMHs increases significantly with age, ranging from approximately 5% in individuals aged 40 years to nearly 100% in those aged 80 years (Zhuang et al., 2018). Severe WMHs have been linked to various negative outcomes, including cognitive impairment (Debette et al., 2019), neuropsychiatric symptoms (Clancy et al., 2021), gait dysfunction (Kim et al., 2016), increased stroke risk (Debette et al., 2019), and poorer stroke recovery (Cheng et al., 2022). However, the underlying mechanisms responsible for the development of WMHs remain incompletely understood, with current research highlighting dysfunctions in cerebral blood flow and the blood–brain barrier as crucial factors (Wardlaw et al., 2019). Previous studies have established an association between intracranial and extracranial atherosclerotic stenosis and white matter hyperintensities (WMHs), although the results of these studies are not consistent (Pu et al., 2009; Lee et al., 2011; Schulz et al., 2013; Park et al., 2015; Duan et al., 2018; Huang et al., 2022).

Atherosclerosis is the primary cause of luminal stenosis in both intracranial and extracranial arteries. Approximately 50% of Asian patients with acute ischemic stroke suffer from intracranial and extracranial atherosclerotic stenosis (Wang et al., 2014). Although WMHs are the primary imaging marker representing damage to the brain’s small vessels, some studies have reported that WMHs were more common in patients with intracranial and extracranial atherosclerotic stenosis (Lee et al., 2011; Park et al., 2015; Duan et al., 2018). A previous meta-analysis demonstrated that carotid artery stenosis was associated with total WMHs, deep WMHs (DWMHs), and periventricular WMHs (PVWMHs) (Ye et al., 2018). However, it is important to note that this meta-analysis only included a limited number of studies (*n* = 8). Furthermore, the study conducted by Ye et al. specifically focused on extracranial internal carotid artery stenosis, and there has been no comprehensive summary of research findings regarding intracranial atherosclerotic stenosis (ICAS) and WMHs. The pathogenic mechanisms and hemodynamic changes of ICAS and extracranial atherosclerotic stenosis (ECAS) are not entirely consistent (Kim et al., 2018). Therefore, it is necessary to determine whether ICAS, similar to ECAS, is related to WMHs. To date, a considerable number of recent studies have been published investigating the correlation between intracranial and extracranial atherosclerotic stenosis and white matter hyperintensities (WMHs) (Duan et al., 2018; Ye et al., 2019; Del Brutto et al., 2020; Fang et al., 2020; Benli et al., 2021; Yin et al., 2021; Choi et al., 2022; Ghaznawi et al., 2022; Huang et al., 2022; Wang et al., 2022). In light of this, we conducted an updated systematic review and meta-analysis to examine the potential association between ICAS or ECAS and WMHs. Additionally, the associations between intracranial and extracranial atherosclerotic stenosis and WMHs in different study designs, severity of atherosclerotic stenosis, WMHs rating method, and WMHs location were further analyzed.

## Methods

This systematic review was carried out based on a predefined protocol (PROSPERO registration number: CRD42023407465), adhering to the Preferred Reporting Items for Systematic Reviews and Meta-Analyses (PRISMA) guidelines (Page et al., 2021).

### Search strategy

The PubMed, Web of Science, and Embase databases were systematically searched from their inception to February 2023. The detailed search formula was as follows: (“white matter hyperintensit*” OR “white matter lesion*” OR “white matter change*” OR “white matter disease*” OR “white matter damage*” OR “leukoaraiosis” OR “leukoencephalopath*” OR “Binswanger’s disease”) AND (“intracranial atherosclero*” OR “cerebral atherosclero*” OR “extracranial atherosclero*” OR “arter* stenosis” OR “intracranial stenosis” OR “extracranial stenosis” OR “carotid stenosis”). The references of the included articles and relevant reviews were manually searched to identify potential studies missed during the initial literature search. Two independent investigators (ZZ and FF) performed a literature, and differences were resolved by a third investigator (WZ) joining the discussion.

### Study selection

This meta-analysis included studies that reported an association between ICAS or ECAS and WMHs in human. The inclusion criteria were as follows: (i) the set criteria for ICAS or ECAS severity was ≥50%; (ii) WMHs were assessed by a quantitative or semiquantitative method based on magnetic resonance imaging (MRI); (iii) the available data for meta-analysis (effect estimates or mean and standard deviation) were reported; and (iv) the article was published in full text in English. The following studies were excluded: (1) studies with unclear criteria for ICAS or ECAS severity; (2) studies with WMHs assessment based on computer tomography (CT); (3) meeting abstracts; and (4) systematic reviews. In the case of duplicated published data, we included the study with the greatest number of participants.

**Figure 1:**
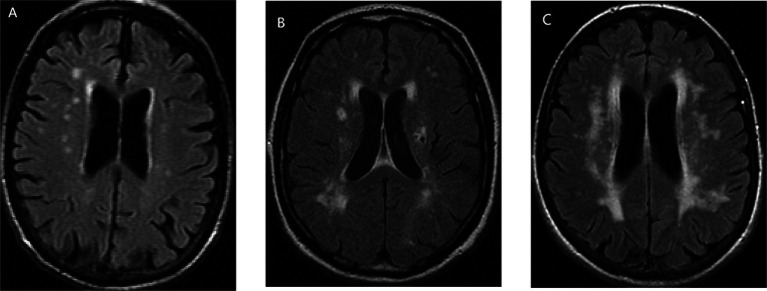
The form of white matter hyperintensities (WMHs), taking Fazekas Scale as an example: **(A)** mild, **(B)** moderate, and **(C)** severe.

ICAS was defined as stenosis in intracranial internal carotid artery, middle cerebral artery, anterior cerebral artery, posterior cerebral artery, intracranial vertebral artery, and basilar artery. ECAS was defined as stenosis in extracranial internal carotid artery, extracranial vertebral artery, external carotid artery, common carotid artery, the proximal portion of subclavian artery, and aortic arch. Imaging examinations to determine the degree of vascular stenosis included Doppler ultrasonography (DUS), CT angiography (CTA), magnetic resonance angiography (MRA), and digital subtraction angiography (DSA). WMHs were defined as hyperintense in the subcortical white matter displayed on T2-weighted sequences (Figure 1) and could be divided into DWMHs and PVWMHs according to their anatomical locations. WMHs within 13 mm from ventricular surface were classified as DWMHs, and WMHs 13 mm or further from the ventricular surface were classified as PVWMHs (Kim et al., 2008). The measurement of WMHs involved quantification methods and semi-quantitative visual rating scales, such as Fazekas Scale (Fazekas et al., 1987), Scheltens Scale (Scheltens et al., 1993), and Age-Related White Matter Changes (ARWMC) Scale (Wahlund et al., 2001).

### Data extraction

Two independent reviewers (ZZ and FF) used a prespecified template to extract information on study characteristics (first author, publication year, country, study design), participant details (sample size, mean age, sex ratio), atherosclerotic stenosis assessment (location, severity, vascular image), WMHs assessment (rating method, location), and statistical analyses (effect estimate and corresponding 95% confidence interval [CI], mean and standard deviation). Relevant missing data were requested by emailing the corresponding authors when possible. Otherwise, the study was not included in the subgroup analysis grouped by missing variables. Effect estimates (usually odds ratios [ORs]) were extracted from the most fully adjusted models. When adjusted effect estimates were not directly provided, we chose unadjusted effect estimates or calculated the ORs using 2 × 2 tables.

### Quality assessment

Two independent reviewers (ZZ and FF) assessed the risk of bias in the study design using the Newcastle-Ottawa scale (Stang, 2010). The checklist consists of three domains (selection, comparability, and exposure) with a total quality score ranging from 0 to 9 points. A score equal to or exceeding 7 indicated a high-quality study. Any disagreements were resolved by a third reviewer (WZ).

### Statistical analysis

Summary measures, including OR and standardized mean difference (SMD), were applied to studies reporting WMHs in the form of categorical and continuous variables, respectively. A random-effects meta-analysis model (DerSimonian Laird method) was used to calculate the pooled OR and SMD (Dersimonian and Laird, 1986). When DWMHs and PVWMHs were reported instead of total WMHs in the included studies, DWMH data was selected for the meta-analysis.

Heterogeneity was assessed using the Cochran Q statistic and was quantified using the *I*^2^ metric. *I*^2^ > 50% was considered statistically significant heterogeneity (Higgins et al., 2003). The source of heterogeneity was investigated via meta-regression (if *n* ≥ 10) and subgroup analyses stratified by multiple different variables. Sensitivity analyses were conducted by excluding one study at a time to examine the robustness of the synthesized results. Publication bias (if *n* ≥ 10) was assessed using funnel plot and Egger’s test (Egger et al., 1997). All statistical tests were two-tailed, and statistical significance was set at *p* < 0.05. Statistical analyses were performed using the R version 4.2.1 software (R Foundation for Statistical Computing, Vienna, Austria).

## Results

### Literature search

**Figure 2:**
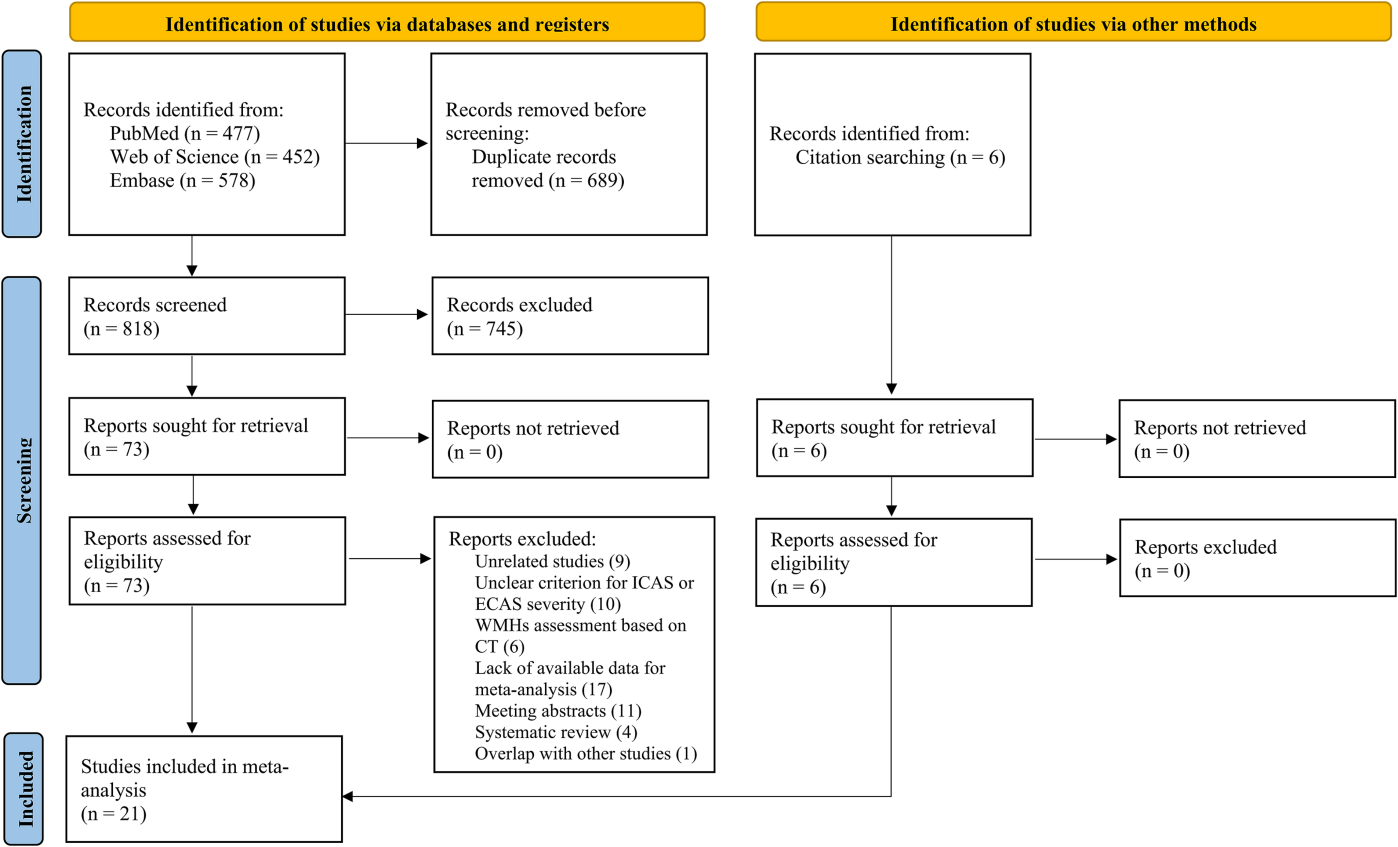
Flowchart presenting the selection of eligible articles.

Figure 2 shows the screening and selection processes of the study. The systematic database search yielded 477, 452, and 578 records from the PubMed, Web of Science, and Embase databases, respectively. After excluding duplicates and reviewing titles and abstracts, 73 articles were considered as potential studies on the association between WMHs and ICAS or ECAS. Following a review of the full texts and a manual search, 21 studies were included in the meta-analyses. Of these, 14 studies were pooled to calculate the OR (Pu et al., 2009; Romero et al., 2009; Muñoz-Cortés et al., 2013; Schulz et al., 2013; Park et al., 2015; Duan et al., 2018; Ye et al., 2019; Del Brutto et al., 2020; Fang et al., 2020; Benli et al., 2021; Yin et al., 2021; Choi et al., 2022; Huang et al., 2022; Wang et al., 2022), and seven studies were pooled to calculate the SMD (Patankar et al., 2006; Chuang et al., 2011; Lee et al., 2011; Cheng et al., 2012; Scherr et al., 2012; Sahin et al., 2015; Ghaznawi et al., 2022).

### Study characteristics

The characteristics of the included studies are summarized in Table 1. Nine studies were conducted in Europe and America, and 12 studies were conducted in East Asia. Five studies compared WMHs in the ipsilateral and contralateral hemispheres of stenotic vessels in patients with ICAS or ECAS, and 16 studies compared WMHs in patients with ICAS or ECAS and controls without ICAS and ECAS. Overall, 21 studies included 10,841 participants (median sample size, *n* = 212: minimum, *n* = 29; maximum, *n* = 2,420). The average age of the participants in the included studies ranged between 51.6 and 72.7 years, and the proportion of females ranged from 8.7 to 81.0%. ICAS were investigated in 11 studies, and ECAS was investigated in 16 studies. The internal carotid artery was the artery of greatest concern (*n* = 20). The criterion for atherosclerotic stenosis was set as greater than 50% in 13 studies, greater than 60% in one study, and greater than 70% in seven studies. Quantitative assessment was conducted in two studies, semi-quantitative assessment in 18 studies, and qualitative assessment in one study. The commonly used scales were Fazekas Scale (*n* = seven), Scheltens Scale (*n* = four), and ARWMC Scale (*n* = three). Total WMHs were investigated in 14 studies, and DWMHs and PVWMHs were investigated in nine studies. According to the quality assessment, approximately half of the studies were of high quality (Supplementary Table S1).

**Table 1:** Study characteristics.

First author, year	Country	Study design	Sample size	Age (mean, y)	Female	Location of atherosclerotic stenosis	Severity of atherosclerotic stenosis	Vascular image	WMHs rating method	WMHs location	NOS
Patankar, 2006	UK	Interindividual	90	NA	NA	Extracranial	≥70%	DUS	Scheltens Scale	Deep and periventricular	5
Pu, 2009	China	Interindividual	185	56.2 ± 10.4	14.1%	Intracranial and extracranial	≥50%	MRA	ARWMC Scale	Total	5
Romero, 2009	USA	Interindividual	1971	58.0 ± 10.0	53.0%	Extracranial	≥50%	DUS	Volumetric	Total	8
Chuang, 2011	China	Intraindividual	106	64.4 ± 8.1	39.6%	Extracranial	≥60%	DUS or MRA	Fazekas Scale	Deep and periventricular	8
Lee, 2011	South Korea	Interindividual	268	67.0 ± 12.4	43.3%	Intracranial and extracranial	≥50%	MRA	Scheltens Scale	Total, deep and periventricular	7
Cheng, 2012	China	Interindividual	43	71.2 ± 6.6	44.2%	Extracranial	≥70%	DUS or MRA	Scheltens Scale	Total	8
Scherr, 2012	Austria	Interindividual	212	70.2 ± 9.1	43.9%	Extracranial	≥50%	DUS	Pantoni Scale	Total	7
Muñoz-Cortés, 2013	Spain	Interindividual	67	53.5 ± 7.0	28.0%	Extracranial	≥50%	DUS	Fazekas Scale	Total	5
Schulz, 2013	UK	Interindividual	671	71.1 ± 11.5	44.3%	Intracranial and extracranial	≥50%	DUS, CTA or MRA	ARWMC Scale	Total	6
Park, 2015	South Korea	Interindividual	697	67.8 ± 12.6	41.8%	Intracranial and extracranial	≥50%	MRA	Fazekas Scale	Deep and periventricular	6
Sahin, 2015	Turkey	Intraindividual	29	68.2 ± 9.2	37.9%	Extracranial	≥70%	CTA, MRA or DSA	Scheltens Scale	Deep and periventricular	8
Duan, 2018	China	Interindividual	2,420	61.9 ± 11.2	32.5%	Intracranial and extracranial	≥50%	MRA	Manolio Scale	Total	5
Ye, 2019	China	Intraindividual	115	67.2 ± 10.2	18.3%	Extracranial	≥70%	DUS	King Scale	Deep and periventricular	9
Del Brutto, 2020	USA	Interindividual	581	71.0 ± 8.4	57.1%	Intracranial	≥50%	MRA	Pantoni Scale	Total	6
Fang, 2020	China	Interindividual	180	63.7 ± 11.6	35.6%	Intracranial	≥70%	MRA	ARWMC Scale	Total	5
Benli, 2021	Turkey	Intraindividual	69	72.7 ± 9.3	50.7%	Extracranial	≥50%	DUS	Fazekas Scale	Deep and periventricular	8
Yin, 2021	China	Interindividual	469	60.2 ± 11.3	27.7%	Intracranial	≥50%	CTA, MRA or DSA	Lesions ≥5 mm in any diameter	Total	6
Choi, 2022	South Korea	Interindividual	1,337	51.6 ± 9.2	13.5%	Intracranial	≥50%	MRA	Fazekas Scale	Deep and periventricular	8
Ghaznawi, 2022	Netherlands	Interindividual	654	57.0 ± 10.0	81.0%	Extracranial	≥70%	DUS	Volumetric	Total	6
Huang, 2022	China	Intraindividual	161	65.7 ± 8.4	8.7%	Intracranial and extracranial	≥50%	DSA	Fazekas Scale	Deep and periventricular	8
Wang, 2022	China	Interindividual	516	59.0 ± 20.0	25.6%	Intracranial	≥70%	MRA	Fazekas Scale	Total	7

ARWMC, age-related white matter changes; CTA, computer tomography; DUS, doppler ultrasonography; DSA, digital subtraction angiography; MRA, magnetic resonance angiography; NA, not applicable; NOS, Newcastle-Ottawa scale; WMHs, white matter hyperintensities.

### Association of ICAS and ECAS with WMHs based on OR

**Figure 3:**
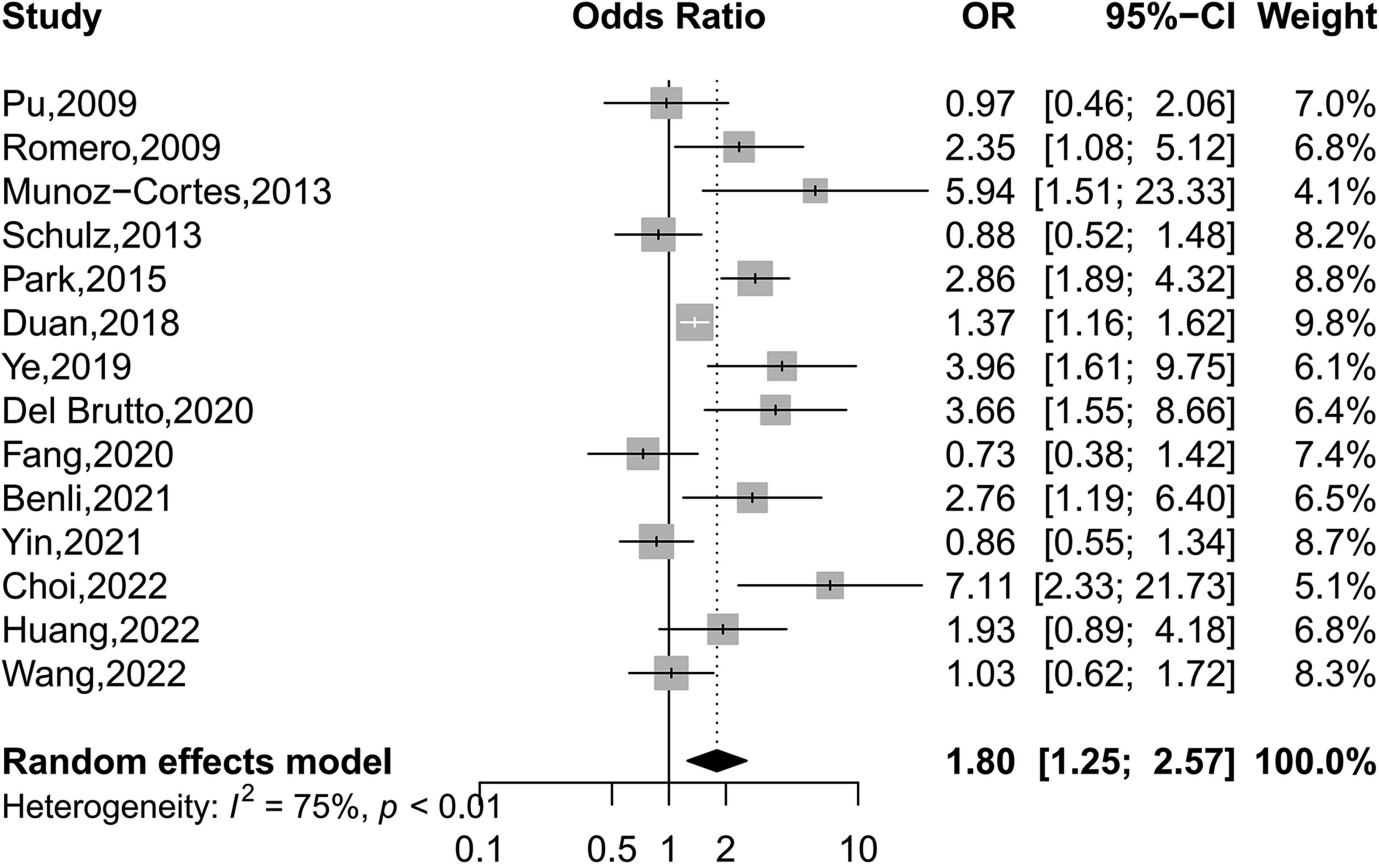
Forest plot depicting intracranial and extracranial atherosclerotic stenosis and the risk of WMHs.

**Figure 4:**
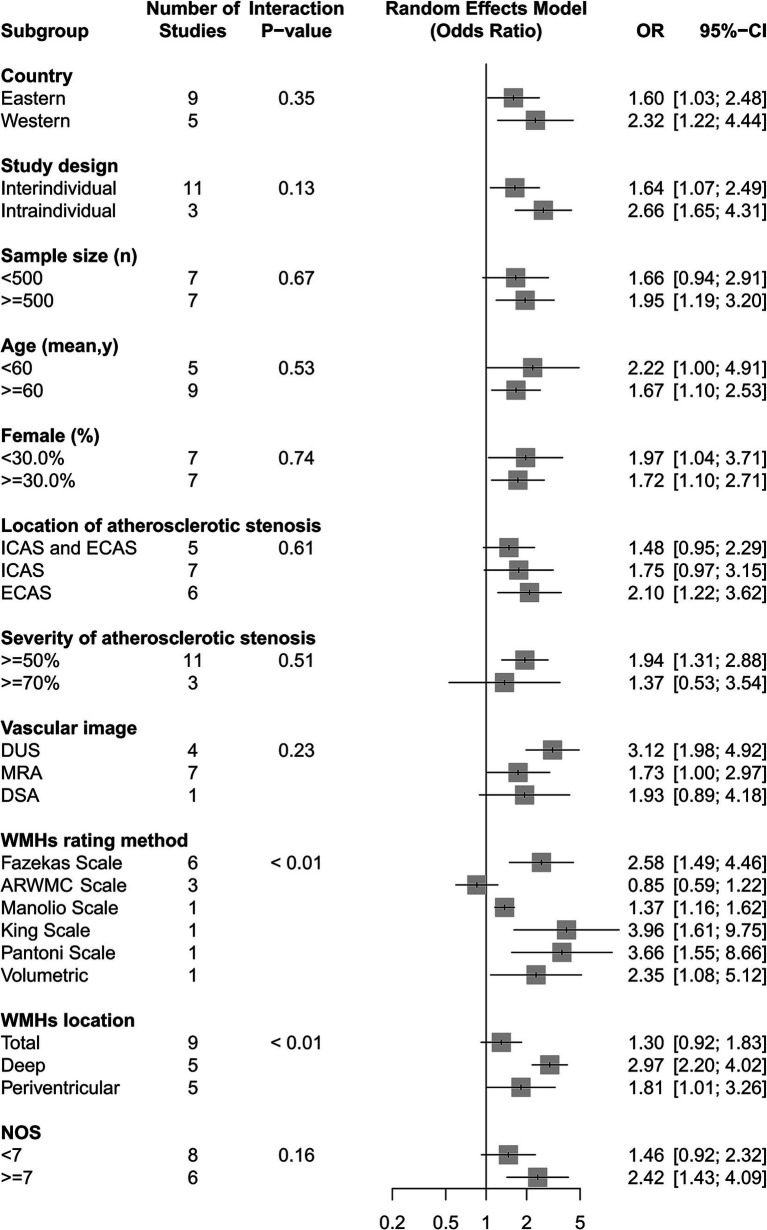
The association of intracranial and extracranial atherosclerotic stenosis with the risk of WMHs in subgroup analyses.

We found intracranial and extracranial atherosclerotic stenosis to be associated with an increased risk of WMHs (OR 1.80, 95% CI 1.25–2.57, *I*^2^ = 75%, 14 studies; Figure 3). Sensitivity analyses further revealed a stable association between intracranial and extracranial atherosclerotic stenosis and WMHs (Supplementary Figure S1). Meta-regression analysis revealed that the source of heterogeneity originated from the WMHs rating method and location (both *p* < 0.05). Subgroup analyses showed that race, age, and sex did not influence this association (Figure 4). Not only patients with intracranial and extracranial atherosclerotic stenosis had increased odds of WMHs compared to controls without intracranial and extracranial atherosclerotic stenosis (OR 1.64, 95% CI 1.07–2.49, *I*^2^ = 77%, 11 studies), but also patients with intracranial and extracranial atherosclerotic stenosis had increased odds of WMHs in the ipsilateral hemisphere of the stenotic vessel compared to the contralateral side (OR 2.66, 95% CI 1.65–4.31, *I*^2^ = 0%, three studies). Intracranial and extracranial atherosclerotic stenosis with a threshold of 50% stenosis was associated with WMHs (OR 1.94, 95% CI 1.31–2.88, *I*^2^ = 76%, 11 studies). According to the location of atherosclerotic stenosis, ECAS (OR 2.10, 95% CI 1.22–3.62, *I*^2^ = 71%, six studies) but not ICAS (OR 1.75, 95% CI 0.97–3.15, *I*^2^ = 84%, seven studies) was associated with WMHs. Both DWMHs (OR 2.97, 95% CI 2.20–4.02, *I*^2^ = 0%, five studies), and PVWMHs (OR 1.81, 95% CI 1.01–3.26, *I*^2^ = 65%, five studies) were associated with intracranial and extracranial atherosclerotic stenosis. WMHs assessed by ARWMC Scale were not associated with intracranial and extracranial atherosclerotic stenosis. The funnel plot (Supplementary Figure S2) for the studies investigating the association between intracranial and extracranial atherosclerotic stenosis and WMHs showed mild asymmetry. However, Egger’s tests (*p* = 0.17) indicated no significant publication bias in this meta-analysis.

### Association of ICAS and ECAS with WMHs based on SMD

**Figure 5:**
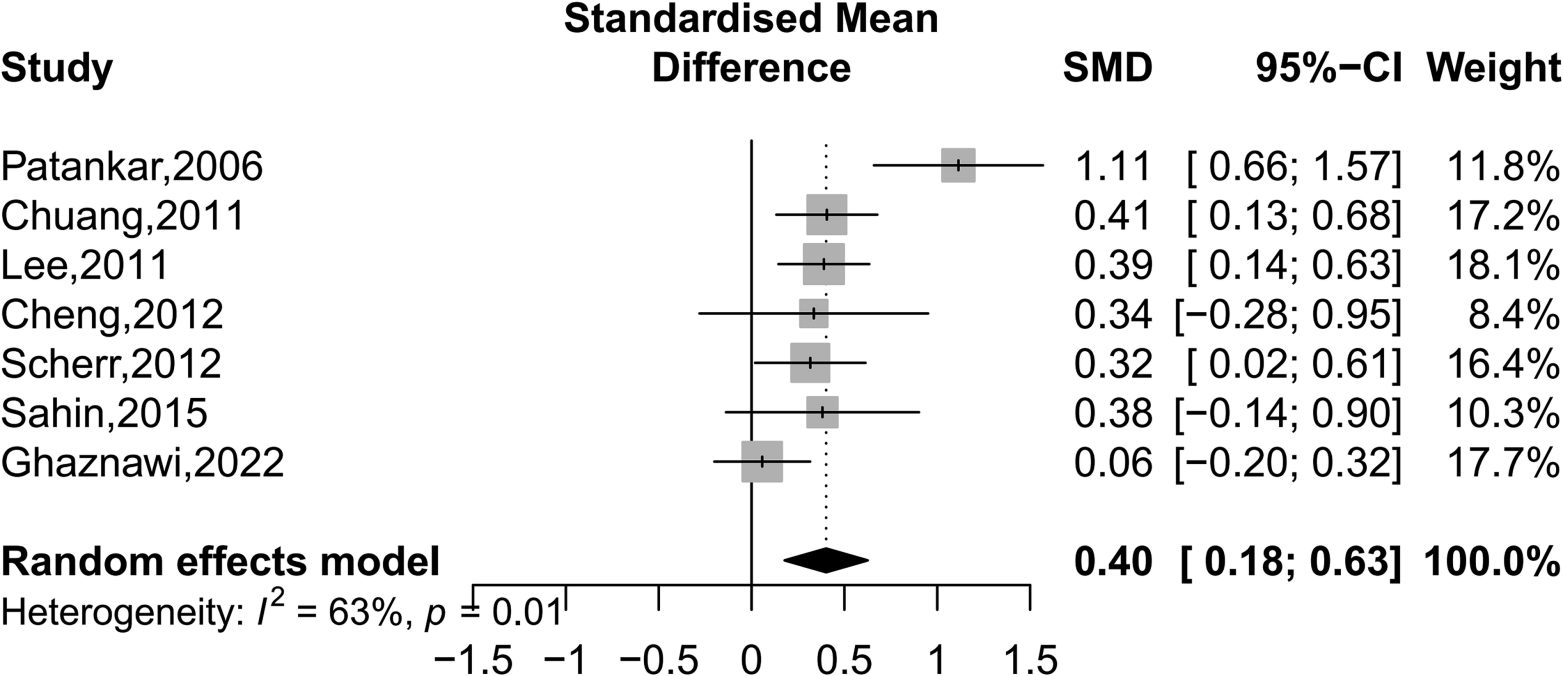
Forest plot depicting intracranial and extracranial atherosclerotic stenosis and the volume of WMHs.

**Figure 6:**
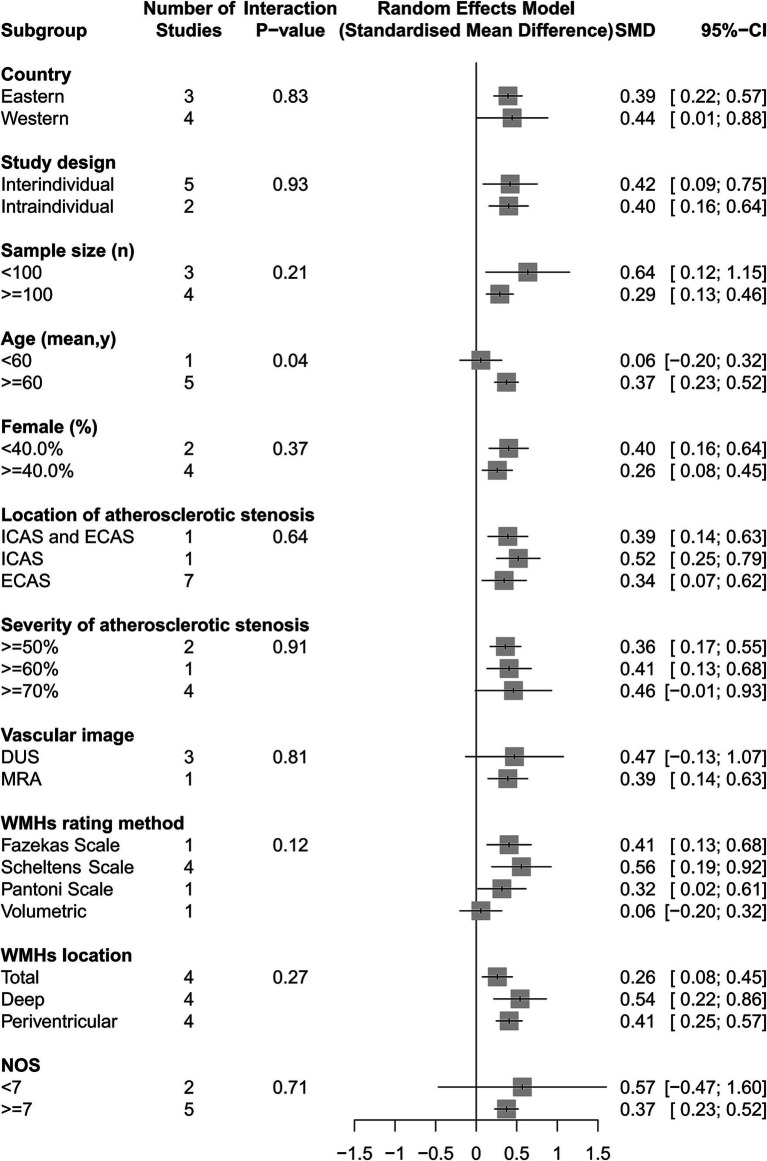
The association of intracranial and extracranial atherosclerotic stenosis with the volume of WMHs in subgroup analyses.

Our analyses found that intracranial and extracranial atherosclerotic stenosis to be associated with increased volume of WMHs (SMD 0.40, 95% CI 0.18–0.63, *I*^2^ = 63%, seven studies; Figure 5). The result remained stable in sensitivity analyses, excluding one study each time (Supplementary Figure S3). Subgroup analyses showed that race and sex did not influence this association (Figure 6). The results were similar between comparison the volume of WMHs in patients with intracranial and extracranial atherosclerotic stenosis and controls without intracranial and extracranial atherosclerotic stenosis (SMD 0.42, 95% CI 0.09–0.75, *I*^2^ = 75%, five studies) and comparison the volume of WMHs in the ipsilateral hemisphere of the stenotic vessel and contralateral side in patients with intracranial and extracranial atherosclerotic stenosis (SMD 0.40, 95% CI 0.16–0.64, *I*^2^ = 0%, two studies). According to the location of atherosclerotic stenosis, both ICAS (SMD 0.52, 95% CI 0.25–0.79, one study) and ECAS (SMD 0.34, 95% CI 0.07–0.62, *I*^2^ = 70%, seven studies) were associated with WMHs. Intracranial and extracranial atherosclerotic stenosis was associated with increased volume of DWMHs (SMD 0.54, 95% CI 0.22–0.86, *I*^2^ = 65%, four studies) and PVWMHs (SMD 0.41, 95% CI 0.25–0.57, *I*^2^ = 0%, four studies). No significant differences in this association were observed between the different semi-quantitative assessment scales.

## Discussion

This systematic review and meta-analysis showed that intracranial and extracranial atherosclerotic stenosis ≥50% was associated with WMHs in deep and periventricular regions. The association was validated in studies designed for both inter-and intra-individual comparisons. Our study showed that ECAS was significantly associated with WMHs, whereas ICAS was only marginally associated with WMHs.

The correlation between ECAS and WMHs confirmed the findings of a previous meta-analysis by Ye et al. (2018). Consistent with previous meta-analysis, the extracranial artery of interest in the included studies was the carotid artery, particularly the extracranial internal carotid artery, which is the main blood vessel supplying the supratentorial white matter. Compared to the study by Ye et al., which only provided SMD values, our study also provided OR values. Our study was conducted to verify the correlation between ICAS and WMHs; however, the results were borderline. Several studies on ICAS and WMHs were excluded because of the lack of data available for meta-analysis, which reported contradictory results, including positive and null associations (Chutinet et al., 2012; Li et al., 2014; Nam et al., 2017; Zhai et al., 2018; Zhao et al., 2019; Ren et al., 2021; Feng et al., 2023). The inconsistencies in the results might be due to the differences in study subjects, sample size, ICAS assessment method, WMHs rating method and statistical analysis between the studies. Overall, several studies with a large sample size (*n* > 1,000) reported similar results, indicating that ICAS was associated with WMHs (Nam et al., 2017; Duan et al., 2018; Zhai et al., 2018; Choi et al., 2022). Another strength of our study is to explore the source of heterogeneity using meta-regression and subgroup analyses. The WMHs rating method was an important source of heterogeneity. Fazekas Scale was suitable for the analysis of WMHs in the form of categorical variable, and Scheltens Scale was suitable for the analysis of WMHs in the form of continuous variable.

The pathophysiology linking intracranial and extracranial atherosclerotic stenosis to WMHs remains unclear. The shared risk factors (e.g., advanced age, hypertension, and diabetes) of intracranial and extracranial atherosclerotic stenosis and cerebral small vessel disease may play a mediating role. However, the intra-individual comparison design which eliminates the influence of vascular risk factors still showed more severe WMHs in the ipsilateral hemisphere of intracranial and extracranial atherosclerotic stenosis than that in the contralateral hemisphere. This indicates that other important mechanisms link intracranial and extracranial atherosclerotic stenosis with WMHs. Cerebral blood flow and cerebrovascular reactivity are impaired in patients with ICAS (Liu and Li, 2016; Yang et al., 2017) or ECAS (Hartkamp et al., 2018), especially in the ipsilateral hemisphere of the stenotic vessel. Endothelial dysfunction (including cerebral blood flow, cerebrovascular reactivity, intracranial pulsatility) is a pivotal pathogenesis of cerebral small vessel disease (Wardlaw et al., 2019). Three studies reported that WMHs were not directly related to arterial stenosis but were independently associated with the stenosis-induced cerebral hemodynamic changes in patients with ICAS (Fang et al., 2020; Ren et al., 2021; Feng et al., 2023). Intracranial and extracranial atherosclerotic stenosis does not necessarily cause decreased cerebral blood flow (Shakur et al., 2014) as it also depends on collateral blood flow, which may result in a null association between intracranial and extracranial atherosclerotic stenosis and WMHs in some previous studies. Recently, high intracranial vascular pulsatility has been linked to the formation of WMHs in individuals with asymptomatic ICAS (Zhao et al., 2022).

Our results showed that the association between intracranial and extracranial atherosclerotic stenosis and WMHs was stronger in the deep white matter than in the periventricular white matter. Neuropathological examination reveals that the main pathological changes are demyelination in DWMHs and the main changes are interstitial edema in PVWMHs (Haller et al., 2013). A cross-sectional study of arterial spin-labeling images showed that ischemia-hypoperfusion is the pathogenesis of DWMHs rather than PVWMHs (Cai et al., 2022). This difference in underlying mechanisms explains the differences in the strength of the association between intracranial and extracranial atherosclerotic stenosis and WMHs at different sites.

Our meta-analysis was based on cross-sectional studies; therefore, causality between intracranial and extracranial atherosclerotic stenosis and WMHs could not be established. A 1 year follow-up study reported no association between 50–69% carotid artery stenosis and ipsilateral WMHs progression (Kwee et al., 2011). Another 7 years follow-up study also found that carotid artery stenosis ≥50% was not associated with WMHs progression, as assessed using the Fazekas Scale (Ihle-Hansen et al., 2021). The only positive findings were observed in a retrospective longitudinal study that reported that ICAS ≥20% was associated with WMHs progression assessed using the modified Rotterdam Progression scale after a 3 years interval (Zhong et al., 2022). A drawback of these studies is that cerebral perfusion was not considered. Reduced cerebral blood flow has been demonstrated to predict WMHs progression in longitudinal studies (Ten Dam et al., 2007; Bernbaum et al., 2015; Promjunyakul et al., 2018). Because cerebral blood flow can be normal in patients with carotid artery stenosis, it is necessary to conduct follow-up studies on specific patients with carotid artery stenosis who have a decrease in cerebral blood flow.

Our study has some limitations. First, gray literature such as meeting abstracts were not included in our meta-analysis. Second, a significant number of studies were excluded due to insufficient data availability, thereby impacting the comprehensiveness of our analysis on the association between ICAS and WMHs. Consequently, further high-quality studies are necessary to conduct a more robust meta-analysis. Third, substantial heterogeneity was observed in the meta-analyses. To address this, we analyzed the data using a random-effect model and explored the heterogeneity using meta-regression. Fourth, some included studies provided univariate analysis results on ICAS or ECAS and WMHs. Thus, the association may be biased due to confounding factors. Finally, a limitation of this study is that it only provides information that intracranial and extracranial atherosclerotic stenosis is associated with WMHs. Further studies are warranted to determine whether improving intracranial and extracranial atherosclerotic stenosis (e.g., stenting) can prevent WMHs progression.

## Conclusion

In summary, despite the considerable heterogeneity and the cross-sectional nature of the included studies, this meta-analysis showed that intracranial and extracranial atherosclerotic stenosis was related to WMHs severity. Notably, this association was found to be significant in ECAS, but less pronounced in ICAS. Future studies should obtain longitudinal data on intracranial and extracranial atherosclerotic stenosis and WMHs progression.

## Data availability statement

The raw data supporting the conclusions of this article will be made available by the authors, without undue reservation.

## Author contributions

WZ conceived and designed the study and wrote the manuscript. WZ, ZZ, and FF performed the literature search and data extraction. FF assisted in data analysis. All authors contributed to the article and approved the submitted version.
