# Supplementary material for: Association between intracranial and extracranial atherosclerosis and white matter hyperintensities: a systematic review and meta-analysis
Source: Front Aging Neurosci. 2024 Jan 8;15:1240509. doi: 10.3389/fnagi.2023.1240509 (PMC10800362; doi:10.3389/fnagi.2023.1240509)
Supplement: Supplementary file 1 [file Data_Sheet_1.PDF]

Supplemental Material

Table S1. The quality assessment of studies by the Newcastle Ottawa scale.

Figure S1. The association of intracranial and extracranial atherosclerotic stenosis with the risk of WMHs in sensitivity analyses.

Figure S2. Funnel plot for the association of intracranial and extracranial atherosclerotic stenosis with the risk of WMHs.

Figure S3. The association of intracranial and extracranial atherosclerotic stenosis with the volume of WMHs in sensitivity analyses.

Table S1. The quality assessment of studies by the Newcastle Ottawa scale.

Study	Selection items				Comparability items		Exposure items			Total score
	Adequate case definition	Represen-tativeness of the cases	Selection of controls	Definition of controls	Age	Vascular risk factors	Ascertai-nment of exposure	Same method of ascertain-ment for cases and controls	Non-Resp onse rate	
Patankar, 2006	1	0	0	0	1	0	1	1	1	5
Pu, 2009	1	0	0	1	0	0	1	1	1	5
Romero, 2009	1	0	1	1	1	1	1	1	1	8
Chuang, 2011	1	0	1	1	1	1	1	1	1	8
Lee, 2011	1	1	0	1	0	1	1	1	1	7
Cheng, 2012	1	1	0	1	1	1	1	1	1	8
Scherr, 2012	1	1	0	1	1	0	1	1	1	7
Muñoz-Cortés, 2013	1	0	0	1	0	0	1	1	1	5
Schulz, 2013	1	1	0	1	0	0	1	1	1	6
Park, 2015	1	1	0	1	0	0	1	1	1	6
Sahin, 2015	1	0	1	1	1	1	1	1	1	8
Duan, 2018	1	0	0	1	0	0	1	1	1	5
Ye, 2019	1	1	1	1	1	1	1	1	1	9
Del Brutto, 2020	1	0	1	1	0	0	1	1	1	6
Fang, 2020	1	0	0	1	0	0	1	1	1	5
Benli, 2021	1	0	1	1	1	1	1	1	1	8
Yin, 2021	1	1	0	1	0	0	1	1	1	6
Choi, 2022	1	1	0	1	1	1	1	1	1	8
Ghaznawi, 2022	1	0	0	1	1	0	1	1	1	6
Huang, 2022	1	0	1	1	1	1	1	1	1	8
Wang, 2022	1	0	0	1	1	1	1	1	1	7

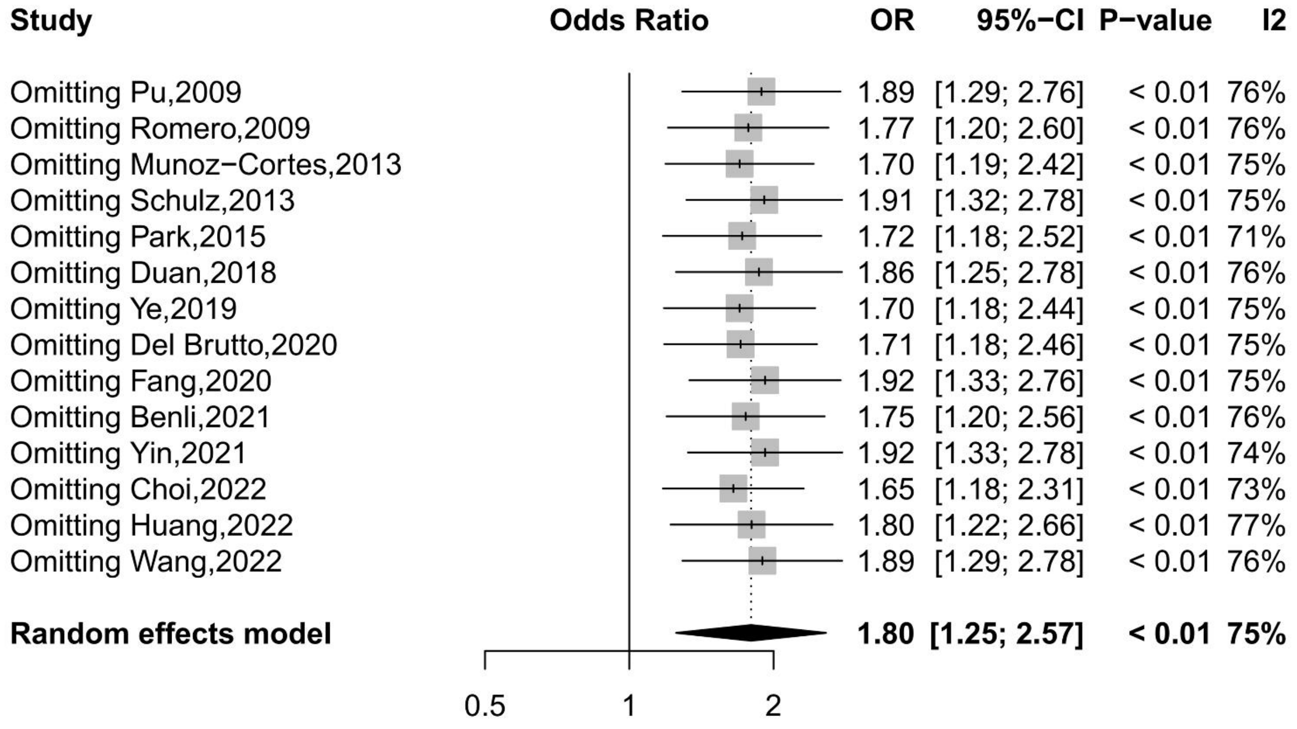

Figure S1. The association of intracranial and extracranial atherosclerotic stenosis with the risk of WMHs in sensitivity analyses.

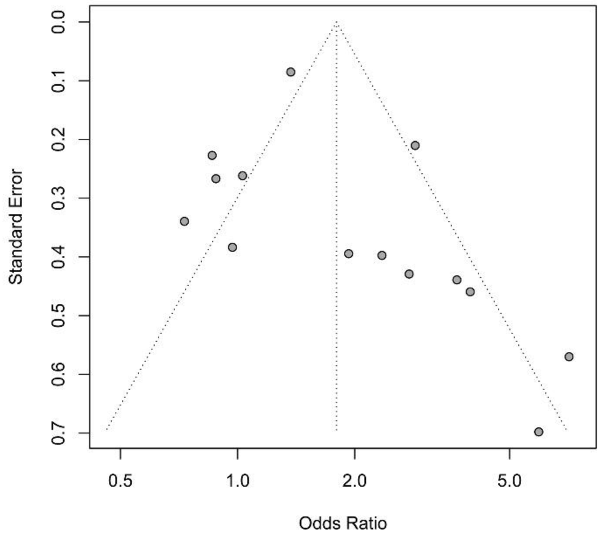

Figure S2. Funnel plot for the association of intracranial and extracranial atherosclerotic stenosis with the risk of WMHs.

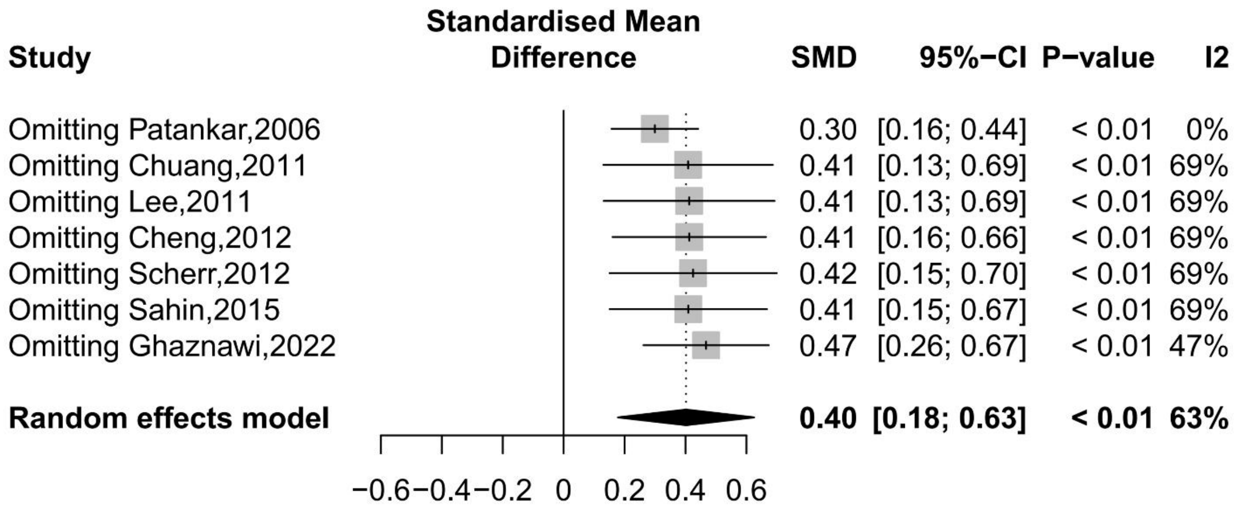

Figure S3. The association of intracranial and extracranial atherosclerotic stenosis with the volume of WMHs in sensitivity analyses.
